# 

**Published:** 1826-01

**Authors:** 


### THE LONDON MEDICAL AND PHYSICAL JOURNAL.

CONTAINING

ORIGINAL CORRESPONDENCE OF EMINENT PRACTITIONERS,

AND

CRITICAL ANALYSIS OF NEW WORKS

RELATING TO MEDICINE, SURGERY, MIDWIFERY, CHEMISTRY, PHARMACY, BOTANY, AND NATURAL HISTORY.

---

EDITED BY

RODERICK MACLEOD, M.D.

LICENTIATE OF THE COLLEGE OF PHYSICIANS OF LONDON;
MEMBER OF THE MEDICO-CHIRURGICAL SOCIETY OF LONDON, AND OF THE ROYAL MEDICAL SOCIETY OF EDINBURGH;
PHYSICIAN TO THE WESTMINSTER GENERAL DISPENSARY;
AND TO THE SCOTTISH HOSPITAL;
LATELY PHYSICIAN TO THE ROYAL INFIRMARY FOR CHILDREN;
LECTURER ON THE PRACTICE OF PHYSIC AND MATERIA MEDICA, AT THE MEDICAL THEATRE, WINDMILL-STREET:

AND

JOHN BACOT, Esq.

MEMBER OF THE ROYAL COLLEGE OF SURGEONS;
SURGEON TO THE ST. GEORGE'S AND ST. JAMES'S DISPENSARY;
AND LATELY SURGEON TO HIS MAJESTY'S GRENADIER REGIMENT OF FOOT GUARDS.

---

VOL. LV.

FROM JANUARY TO JUNE, 1826.

Et quoniam variant morbi, variabimus artes;

Mille mali species, mille salutis erunt.

---

45588

*LONDON:*

PRINTED FOR THE PROPRIETORS,

*By J. and C. Adlard, Bartholomew Close;*

PUBLISHED BY J. SOUTER, 73, ST. PAUL'S CHURCH-YARD;

AND MAY BE HAD OF ALL BOOKSELLERS.

### NOTICE TO CORRESPONDENTS.

*The Communications of* Mr. Jewel, Mr. Clay, Mr. Boyle, Mr. Norman, Mr. Blackett, *and* Dr. Stoker, *have been received.*

☞ *We are compelled to omit the* List of Books *from want of room.*

### Errata.

In the Index .................... *for* Chloruret *read* Chloride.

